# Frequency and correlates of suicidal ideation in preoperative cardiac surgery patients

**DOI:** 10.1192/j.eurpsy.2021.2192

**Published:** 2021-08-13

**Authors:** O. Nikolaeva, E. Nikolaev, D. Hartfelder, E. Lazareva, S. Petunova, N. Grigorieva

**Affiliations:** 1 Cardiosurgery, Republic Cardiology Clinic, Cheboksary, Russian Federation; 2 Medical Faculty, Ulianov Chuvash State University, Cheboksary, Russian Federation; 3 Social And Clinical Psychology, Ulianov Chuvash State University, Cheboksary, Russian Federation

**Keywords:** Suicidal ideation, Suicidal risk, time perspective, cardiac surgery patients

## Abstract

**Introduction:**

Depressive disorders are common for cardiac patients; however, a surgical intervention enhances their distress. How typical is suicidal ideation for cardiac surgery patients and with what clinical and psychological signs does it correlate?

**Objectives:**

To estimate the frequency of suicidal ideation and correlations between suicidal ideation, clinical and psychological manifestations in cardiac surgery patients.

**Methods:**

We examined 60 cardiac surgery patients, aged 25 – 65, prior to their operation. The Pierson correlation between manifestation of suicidal ideation, clinical and psychological signs was calculated with p<0.05.

**Results:**

We revealed suicidal ideation in 3.33% of cardiac surgery patients. Its intensity credibly correlated with the frequency of taking alcohol (r=.32), as well as with manifestation of dysorexia (r=.59), dissatisfaction with life (r=.53), general level of depression (r=.49), sleeping disorders (r=.44), sense of guilt (r=.43), asthenia (r=.31), self-abhorrence (r=.29), and irritability(r=.29). A higher level of suicidal ideation correlated with a lower index of Positive-Past in their personal time perspective (r=-.27), which revealed itself in a patient having lack of positive impressions and recollections of their past life, which reduced a person’s adaptability in the present.

**Conclusions:**

The frequency of suicidal ideation in preoperative cardiac surgery patients is not high. Nevertheless, we should bear in mind that high suicidal risk is characteristic for patients with not only depression, but also alcohol problems, as well as for those who have manifestations of negative attitude to their past.

**Disclosure:**

No significant relationships.

